# Socioeconomic inequalities in prevalence, awareness, treatment and control of hypertension: evidence from the PERSIAN cohort study

**DOI:** 10.1186/s12889-022-13444-x

**Published:** 2022-07-22

**Authors:** Mahin Amini, Mahdi Moradinazar, Fatemeh Rajati, Moslem Soofi, Sadaf G. Sepanlou, Hossein Poustchi, Sareh Eghtesad, Mahmood Moosazadeh, Javad Harooni, Javad Aghazadeh-Attari, Majid Fallahi, Mohammad Reza Fattahi, Alireza Ansari-Moghaddam, Farhad Moradpour, Azim Nejatizadeh, Mehdi Shahmoradi, Fariborz Mansour-Ghanaei, Alireza Ostadrahimi, Ali Ahmadi, Arsalan Khaledifar, Mohammad Hossien Saghi, Nader Saki, Iraj Mohebbi, Reza Homayounfar, Mojtaba Farjam, Ali Esmaeili Nadimi, Mahmood Kahnooji, Farhad Pourfarzi, Bijan Zamani, Abbas Rezaianzadeh, Masoumeh Ghoddusi Johari, Masoud Mirzaei, Ali Dehghani, Seyed Fazel Zinat Motlagh, Zahra Rahimi, Reza Malekzadeh, Farid Najafi

**Affiliations:** 1 Behavioral Disease Research Center, Kermanshah University of Medical Sciences, Kermanshah, Iran; 2 Department of Health Education and Promotion, Research Center for Environmental Determinants of Health, School of Health, Kermanshah University of Medical Sciences, Kermanshah, Iran; 3 Health Institute, Kermanshah University of Medical Sciences, Kermanshah, Iran; 4 Digestive Disease Research Center, Digestive Diseases Research Institute, Tehran University of Medical Sciences, Tehran, Iran; 5 Liver and Pancreatobiliary Diseases Research Center, Digestive Diseases Research Institute, Tehran University of Medical Sciences, Tehran, Iran; 6 Gastrointestinal Cancer Research Center, Non-Communicable Diseases Institute, Mazandaran University of Medical Sciences, Sari, Iran; 7 Social Determinants of Health Research Center, Yasuj University of Medical Sciences, Yasuj, Iran; 8 Clinical Research Institute,Occupational Medicine Center, Social Determinants of Health Research Center, Urmia University of Medical Sciences, Urmia, Iran; 9 Department of Occupational Health Engineering, School of Public Health, Non Communicable Disease Research Center, Sabzevar University of Medical Sciences, Sabzevar, Iran; 10 Gastroenterohepatology Research Center, Shiraz University of Medical Sciences, Shiraz, Iran; 11 Health Promotion Research Center, Zahedan University of Medical Sciences, Zahedan, Iran; 12 Social Determinants of Health Research Center, Research Institute for Health Development, Kurdistan University of Medical Sciences, Sanandaj, Iran; 13 Molecular Medicine Research Center, Hormozgan University of Medical Sciences, Bandar Abbas, Iran; 14 Endocrinology and Metabolism Research Center, Hormozgan University of Medical Sciences, Bandar Abbas, Iran; 15 Gastrointestinal and Liver Diseases Research Center, Guilan University of Medical Sciences, Rasht, Iran; 16 Nutrition Research Center, Tabriz University of Medical Sciences, Tabriz, Iran; 17 Modeling in Health Research Center, Shahrekord University of Medical Sciences, Shahrekord, Iran; 18 Hearing Research Center, Department of Otolaryngology, Head and Neck Surgery, Clinical Sciences Research Institute, Ahvaz Jundishapur University of Medical Sciences, Ahvaz, Iran; 19 NonCommunicable Diseases Research Center, Fasa University of Medical Sciences, Fasa, Iran; 20 Non-Communicable Diseases Research Center, Rafsanjan University of Medical Sciences, Rafsanjan, Iran; 21 Digestive Disease Research Center, Ardabil University of Medical Sciences, Ardabil, Iran; 22 Colorectal Research Center, Shiraz University of Medical Sciences, Shiraz, Iran; 23 Breast Diseases Research Center, Shiraz University of Medical Sciences, Shiraz, Iran; 24 Yazd Cardiovascular Research Centre, Shahid Sadoughi University of Medical Sciences, Yazd, Iran; 25 Centre For Healthcare Data Modeling, School of Public Health, Shahid Sadoughi University of Medical Sciences, Yazd, Iran; 26 Hearing Research Center, Department of Biostatistics and Epidemiology, School of Public Health, Clinical Sciences Research Institute, Ahvaz Jundishapur University of Medical Sciences, Ahvaz, Iran; 27 Digestive Diseases Research Institute, Tehran University of Medical Sciences, Tehran, Iran; 28 Department of Epidemiology, School of Health, Research Center for Environmental Determinants of Health, Research Institute for Health, Kermanshah University of Medical Sciences, Kermanshah, Iran

**Keywords:** Hypertension, Inequality, Awareness, Treatment, Control, PERSIAN Cohort

## Abstract

**Background:**

Elevated blood pressure is associated with cardiovascular disease, stroke and chronic kidney disease. In this study, we examined the socioeconomic inequality and its related factors in prevalence, Awareness, Treatment and Control (ATC) of hypertension (HTN) in Iran.

**Method:**

The study used data from the recruitment phase of The Prospective Epidemiological Research Studies in IrAN (PERSIAN). A sample of 162,842 adults aged >  = 35 years was analyzed. HTN was defined according to the Joint National Committee)JNC-7(. socioeconomic inequality was measured using concentration index (Cn) and curve.

**Results:**

The mean age of participants was 49.38(SD =  ± 9.14) years and 44.74% of the them were men. The prevalence of HTN in the total population was 22.3%(95% CI: 20.6%; 24.1%), and 18.8%(95% CI: 16.8%; 20.9%) and 25.2%(95% CI: 24.2%; 27.7%) in men and women, respectively. The percentage of awareness treatment and control among individuals with HTN were 77.5%(95% CI: 73.3%; 81.8%), 82.2%(95% CI: 70.2%; 81.6%) and 75.9%(95% CI: 70.2%; 81.6%), respectively. The Cn for prevalence of HTN was -0.084. Two factors, age (58.46%) and wealth (32.40%), contributed most to the socioeconomic inequality in the prevalence of HTN.

**Conclusion:**

The prevalence of HTN was higher among low-SES individuals, who also showed higher levels of awareness. However, treatment and control of HTN were more concentrated among those who had higher levels of SES, indicating that people at a higher risk of adverse event related to HTN (the low SES individuals) are not benefiting from the advantage of treatment and control of HTN. Such a gap between diagnosis (prevalence) and control (treatment and control) of HTN needs to be addressed by public health policymakers.

## Introduction

To obtain the proposed Sustainable Development Goals (SDGs) and targets, many countries have focused on advancing universal health coverage as their essential health policy [1]. One of the SDGs targets is a 30% reduction in premature mortality from non-communicable diseases (NCD) by 2030. This is mainly accomplished by disease prevention and treatment [2].

Hypertension (HTN) is one of the most important risk factors for some NCD such as cardiovascular diseases, stroke, and chronic kidney disease. It is estimated to cause 12.8% of all-cause mortality and 57 million disability adjusted life years (DALY) [3–7]. Yet, many individuals are often unaware of having HTN, especially at its initial phases, due to a lack of specific clinical signs and do not seek treatment and control of HTN; therefore, its detection in the community is usually delayed [8].

Iranians with HTN are 1.35 times more likely to develop premature coronary artery disease [9]. Studies conducted in different geographic areas of Iran have indicated that HTN prevalence ranges from 4.5% to 46.9%. Results of a meta-analysis conducted over 2003–2018 has shown that prevalence, awareness, treatment, and control (ATC) of HTN in Iran are 20.4%, 49.3%, 44.8%, 37.4%, respectively [10]. However, Iran has achieved a good improvement in management of HTN in recent years [11].

Differences in health conditions between socioeconomic groups leads to inequality in health and this, in turn, is one of the major public health issues worldwide [12, 13]. Socio-economic status (SES) has been proven as a major risk factor driving health inequity [14]. Prevalence of HTN and its ATC have been reported to differ by socioeconomic disparities in Portugal and Netherlands [15, 16]. However, conflicting results have been shown in the effects of socioeconomic determinants on the prevalence of HTN. Although the prevalence of HTN is more among the higher socioeconomic status levels in some studies in different settings [17–20], other studies have shown the reverse effect [21–23]. In Indonesia, socioeconomic status has differential impact on the detection of HTN and in taking medications [24]. In fact, some studies have shown that individuals from richest groups were more likely to be hypertensive, had higher awareness of their condition, were more likely to receive treatment, and had controlled HTN, compared to their counterparts [25–27].

Previous studies reported the prevalence, treatment and control of HTN regionally in Iran [28, 29]. To our knowledge no evidence from national representative data are available regarding the SEI in prevalence and ATC of HTN in Iran. Therefore, the aim of this study is to examine the SEI in the HTN burden and its management including ATC among Iranians aged 35 years and above, using data from 18 geographically distinct cohort centers throughout Iran.

## Methods

### Data and study setting

In this study, data from the recruitment phase of the Prospective Epidemiological Research Studies in IrAN (PERSIAN), a cohort study including individuals from 18 regions with different ethnicities and cultures, was used. The PERSIAN cohort initiated in 2014 and aimed to discover the potential socioeconomic, environmental, behavioral, and para-clinical risk factors of common NCD in Iran. In each of the PERSIAN Cohort centers, between 5,000 and 20,000, in total about 163,770 individuals aged 35–70 years, from urban and rural areas have been enrolled. Using the records for each family in public health system, the study team at each center did a dedicated census and a door-to-door survey of all residents in urban areas to register the home addresses. However, in the rural area, local health units had all required information. Finally using a stratified (by place of living in urban or rural areas) random sampling, the recruited people were invited to the cohort centers. More information about this study can be found at https://persiancohort.com/ and previously published PERSIAN Cohort protocol [30, 31].

### Data collection and measurements

All data and measurements in the PERSIAN cohort centers were collected following the same protocols and standard equipment for consistency of results. Electronic questionnaires in three main categories: general (including questions on demographic variables, socio-economic status and other questions on lifestyle), medical and nutrition, were completed by trained and experienced interviewers.

### Blood pressure measurement

The main outcomes in this study are the prevalence and ATC of HTN. For all individuals, blood pressure was measured twice in both arms in the sitting position and after a ten-minute rest. The average of the second measurement in both arms was used as the systolic and diastolic pressures. To diagnose high blood pressure, the Joint National Committee on Prevention, Detection, Evaluation, and Treatment of HTN (JNC-7) classification was used [32]. Accordingly, individuals with a systolic blood pressure of 140 mmHg or more, and/or a diastolic blood pressure of 90 mmHg or more were considered to be hypertensive. Those taking antihypertensive medications were also considered to have HTN.

To assess people's awareness of HTN among those with high blood pressure, after measuring and confirming HTN, individuals were asked if they were aware of having HTN diagnosed by a physician. To find out if people who are aware of their HTN are being treated, their medications were checked and if they were taking antihypertensive drugs, they were considered as individuals receiving treatment; in case of a self-reported use of antihypertensive medication, those individuals were also considered to be receiving treatment. Among the participants treated with antihypertensive medications, if the blood pressure was below 140/90 mmHg, it was considered as controlled blood pressure [33].

Body mass index (BMI) was calculated as weight (kg) divided by height (m^2^). Individuals with a BMI less than 25 kg/m^2^ were categorized as normal, between 25.0 and 29.9 kg/m^2^ as overweight, between 30–34.9 as first-degree obesity and equal to or more than 35 as second degree obesity [34].

In this study, people who smoked less than 100 cigarettes in their lifetime were in the non-smoking group, and those who smoked more than 100 cigarettes in the past but do not currently smoke, were considered as former smokers; people who smoked more than 100 cigarettes in their lifetime and were smoking at the time of data collection, were in the smokers group [35]. Alcohol consumption was measured by asking about the amount, frequency and duration of consumption of any alcoholic beverages (wines, beers, and spirits) in each age. Then the participants were categorized to ever and never used. The same questions were asked about the substance abuse. For the purpose of this study, we also categorized the people as ever and never used. Hookah use was also measured by asking individuals about their full history of use as well as the frequency of use.

In this article Multicollinearity between all variables has been checked with VIF (Variable Inflation Factors). VIF determines the strength of the correlation between the independent variables. VIF of 5 and above indicates a multicollinearity problem.

### Statistical analyses

Prevalence of HTN, proportion of ATC were calculated. Given the cluster sampling design of the study, survey design was used for estimating the prevalence and proportions. We used centers as the primary sampling units in the survey design and used probability weights, defined as the inverse probability of being selected in the survey at the district level based on data of the national census in 2016. For all estimates, we reported 95% confidence intervals. Data were analyzed using Stata software (version 14.1) (Stata Corp, College Station, TX, USA).

### Measurement of socioeconomic status

In order to determine the SES of participants, the main asset-based wealth index method for all cohort centers was used. Wealth score index is estimated by multiple correspondence analysis (MCA) of the following variables: access to a freezer, access to a washing machine, access to a dish washer, access to a computer, access to internet, access to a motorcycle, access to a car (no access, access to a car with price of < 500 million Rials, and access to a car with price of > 500 million Rials), ( 1US$ was approximately equivalent to 25,940 Rials in 2014), access to a vacuum cleaner, color TV type (no color TV or regular color TV vs. Plasma color TV), owning a mobile, owning a PC or laptop, international trips in lifetime (never, just pilgrimage, both pilgrimage or non-pilgrimage trips. SES was categorized into (*a*) first quantile (poorest); (*b*) second quantile; (*c*) third quantile; (*d*) fourth quantile; (*e*) five quantile (richest).

### Inequalities measurement

For the purpose of this study, SEI was measured using the concentration index and concentration curve [36, 37]. The concentration curve depicts the cumulative percentage of HTN (y-axis) against the cumulative percentage of the population, ranked by asset (x-axis) from the poorest to the richest. Then concentration index was defined as twice the area between the concentration curve and line of equality. It was computed as twice the covariance of the prevalence of HTN and a person's relative rank in terms of economic status, divided by the variable mean. The numerical value of the concentration index is between -1 and + 1. The number zero for the concentration index on the curve corresponds to the ˚45 line (line of equality), which indicates the complete equality in the distribution of the given variable in various socioeconomic groups. If the numerical value of the index is positive, the curve lies below the line of equality, which means that the prevalence of the given variable is higher in people with high socioeconomic status, and vice versa.

Concentration index calculated according to Formula 1.

1 $$\mathrm{CI}= \frac{2}{\overline{\mathrm{Y}}}\mathrm{COV }(\mathrm{Yi}.\mathrm{Ri})$$

Where $\overline{\mathrm{Y} }$ is the average health variable in the total population and R_i_ represents the rank of each person according to the socioeconomic quintiles (for the poorest person R_1_ = 1/N and for the richest person is equal to R_5_ = N/N). Y_i_ is a health variable for i. For binary variables, the concentration index may not be in the range of -1 to + 1. To solve this problem, Wagstaff and Erreygers have proposed two different methods of normalization. In this study, the normalized concentration index was used by Wagstaff method according to Formula 2 [38, 39].

2 $${\mathrm{C}}_{\mathrm{n}}=\mathrm{CI}/1-\upmu$$

3 $${\mathrm{C}}_{\mathrm{n}}=\frac{\sum_{\mathrm{k}}\left(\frac{{\upbeta }_{\mathrm{k}}{\overline{\mathrm{x}} }_{\mathrm{k}}}{\upmu }\right){\mathrm{C}}_{\mathrm{k}}}{1-\upmu }+\frac{{\mathrm{GC}}_{\upvarepsilon }/\upmu }{1-\upmu }$$

$\overline{X }$_k_ represents the mean of each of the explanatory variables, C_K_ indicates the value of the concentration index for the explanatory variable that has been normalized. Due to the binary of the dependent variable in this study in this formula, ${\beta }_{k}$ is the marginal effect taken into account from the logistics model for each variable. All variables are entered into the model under stepwise predictor selection. The elasticity of each variable is calculated by the formula $\frac{{\beta }_{k}{\overline{x} }_{k}}{\mu }$ Elasticity; sensitivity or responsiveness of the dependent variable to the explanatory variable, for example, indicates that if one percent of the explanatory variable changes, how many percent of the dependent variable changes. $\frac{\frac{G{C}_{\varepsilon }}{\mu }}{1-\mu }$ is called the generalized concentration index or the residual component. In this study, we decomposed the concentration index only for the prevalence of HTN in the population to the factors contributed in inequality.

In this study, we show the concentration index for the dependent variable with C_n_ and for the independent variables with C_k_.

Missing data, which were less than 1%, were excluded from the study. Finally, 162,842 men and women from all cohort centers were analyzed to determine the prevalence of HTN and ATC and to calculate the concentration index. *P*-value < 0.05 was determined for statistical significance. All data were analyzed with Stata software version 15 and Excel 2016 software using appropriate statistical tests.

## Results

### Descriptive results

From 163,770 PERSIAN Cohort participants (and after exclusion of 928 people with missing information on measurement of blood pressure), 44.74% were men. The mean age of all participants in the study was 49.38(SD =  ± 9.14) years and was similar in both sexes. The number of participants with HTN was 41,266 (22.3%). Of the illiterate participants, 40.84% were hypertensive compared to 15.42% of individuals having a college degree. Among all participants, 23.31% were overweight and 8.18% were obese. The prevalence of HTN among these two groups were 29.57% and 38.76%, respectively. From all hypertensive individuals, 77.5% were aware of their HTN and 82.2% received treatment. Among those who were aware of their condition, 97.33% were treated, and among those who were treated, 75.9% had controlled HTN (Table 1). The mean systolic blood pressure of all participants was 112.20 (SD =  ± 17.18) mmHg and mean diastolic Blood pressure was 71.73 (SD =  ± 11.08) mmHg.

**Table 1:** Prevalence, awareness, treatment and control of hypertension based on the JNC7 hypertension guideline^a^

Variables		Total (%)	HTN Prevalence^b^ (95%CI)	Awareness^c^ (95%CI)	Treatment^d^ (95%CI)	Controlled^e^ (95%CI)
Total (%)		162,842(100%)	41,266(22.3)	25,788(77.5)	33.707(82.2)	18,495(75.9)
Sex	male	72,861(44.74)	18.85(16.87,20.99)	60.95(55.32,66.31)	72.51(65.08,78.88)	72.17(66.44,77.26)
	Female	89,981(55.26)	25.92(24.23,27.68)	83.97(80.87,86.65)	89.51(86.29,92.05)	75.84(70.77,80.27)
Age	35–39	27,440(16.85)	5.91(4.93,7.06)	45.19(39.46,51.06)	62.55(52.72,71.44)	79.58(74.57,83.82)
	40–44	30,254(18.58)	10.54(8.99,12.31)	58.33(51.80,64.57)	69.79(62.68,76.06)	77.05(71.48,81.81)
	45–49	29,289(17.99)	18.37(16.33,20.60)	67.92(61.81,73.48)	76.44(69.93,81.90)	75.61(70.77,79.87)
	50–54	25,857(15.88)	28.39(25.44,31.53)	74.60(69.45,79.15)	82.56(77.21,86.87)	75.36(70.57,79.60)
	55–59	22,980(14.11)	37.68(34.88,40.56)	78.73(75.14,81.93)	86.28(82.18,89.56)	73.71(67.49,79.11)
	> 59	27,022(16.59)	52.10(49.31,54.87)	81.55(78.88,83.95)	89.12(85.84,91.71)	73.08(67.60,77.94)
Education	Illiterate	33,549(20.61)	40.84(37.07,44.72)	82.83(79.98,85.35)	88.14(84.74,90.86)	70.63(64.35,76.21)
	1–5 y	51,797(31.83)	24.16(21.41,27.15)	76.15(71.66,80.13)	83.97(79.28,87.77)	75.36(70.18,78.57)
	6-8y	23,053(14.16)	19.32(16.79,22.13)	68.05(61.55,73.92)	78.16(70.83,84.06)	74.34(69.25,78.84)
	9-12y	34,989(21.50)	15.84(13.96,17.93)	66.26(60.45,71.62)	76.91(69.98,82.64)	77.42(72.40,81.76)
	≥ 13 y	19,362(11.90)	15.42(13.55,17.49)	64.91(57.77,71.43)	76.65(67.26,83.98)	77.18(71.24,82.20)
Marital status	Married	148,270(91.05)	21.58(19.89,23.37)	72.58(68.12,76.63)	81.45(76.14,85.80)	74.38(69.35,78.83)
	Single	3416(2.10)	8.73(6.54,11.57)	42.73(35.50,50.29)	51.84(42.76,60.81)	68.29(57.90.77.13)
	divorced	11,156(6.85)	39.57(37.14,42.05)	87.13(84.98,89.02)	91.58(88.66,93.80)	75.14(68.09,81.07)
Hookah	No	150,107(92.18)	22.54(20.77,24.43)	74.60(70.27,78.49)	82.83(77.72,86.96)	74.54(69.23,79.20)
	Yes	12,735(7.82)	20.11(17.64,22.83)	64.17(57.39,70.43)	75.35(67.77,81.63)	73.16(68.96,76.98)
Drug abuse	No	146,330(89.86)	22.57(20.72,24.53)	75.19(71.01,78.94)	82.92(77.81,87.05)	74.89(69.57,79.56)
	Yes	16,476(10.12)	20.51(18.72,22.42)	62.40(55.97,68.42)	76.56(69.96,82.07)	70.55(66.21,74.55)
Alcohol	No	152,367(93.57)	22.70(20.98,24.52)	74.81(70.86,78.40)	83.06(78.27,86.97)	74.62(69.37,79.24)
	Yes	10,435(6.41)	17.45(15.16,20.02)	56.61(47.51,65.29)	68.26(57.15,77.62)	71.01(65.02,76.35)
Smoking status	No	127,431(78.25)	23.23(21.43,25.12)	76.54(72.47,80.16)	83.73(78.96,87.59)	74.26(69.9,78.91)
	Current	22,928(14.08)	14.71(13.05,16.54)	60.91(52.59,68.64)	74.42(65.23,81.86)	75.77(70.32,80.49)
	Former	12,483(7.67)	28.12(25.72,30.65)	64.85(60.65,68.83)	77.97(72.89,82.33)	74.68(69.75,79.05)
BMI	> 25	44,954(27.71)	12.61(11.21,14.15)	63.04(59.03,66.88)	77.39(72.01,81.99)	76.27(71.55,80.42)
	25.0–29.9	66,181(40.80)	21.83(19.40,24.48)	72.32(67.72,76.48)	80.94(75.33,85.52)	75.23(69.65,80.08)
	30.0–34.9	37,813(23.31)	29.57(26.85,32.45)	77.59(73.82,80.95)	84.55(79.82,88.33)	74.68(69.09,79.56)
	≥ 35	13,261(8.18)	38.76(35.18,42.46)	81.64(78.16,84.67)	86.47(81.64,90.18)	70.34(63.67,76.24)
Economic status	1^st^ quintile	32,562(20.05)	27.83(24.24,31.73)	76.68(72.83,80.14)	83.08(78.81,86.63)	70.41(765.09,75.23)
	2^nd^ quintile	34,543(21.27)	24.44(22.02,27.03)	75.46(71.23,79.24)	83.21(78.86,86.82)	73.39(69.26,77.15)
	3^rd^ quintile	33,404(20.56)	22.45(20.59,24.44)	74.77(70.69,78.46)	82.60(78.0,86.42)	74.74(69.04,79.70)
	4^th^ quintile	35,354(21.76)	19.53(17.47,21.78)	70.01(64.28,75.18)	80.07(73.09,85.60)	76.03(70.16,81.05)
	5^th^ quintile	26,574(16.36)	19.45(17.18,21.93)	71.72(65.46,77.24)	82.27(73.89,88.38)	77.84(71.24,83.28)

^a^For all calculations we used centers as the primary sampling units in the survey design and used probability weights

^b^ Prevalence rate is calculated by dividing people with HTN to the total population

^c^ Awareness is calculated by dividing people who are aware of their HTN into the total number of people with HTN

^d^Treatment is calculated by dividing people who have received antihypertensive drugs into people who are aware of their HTN

^e^ Control is calculated by dividing people with normal HTN who have been treated with antihypertensive drugs over the total number of people treated with antihypertensive drugs

The prevalence of HTN in people who use hookah, drugs, and alcohol was less than those who did not. But the prevalence of HTN in former smokers was higher than in current smokers and none smokers.

### Contributing factors related to the prevalence and ACT of HTN

In univariate analysis people with hypertension and better awareness to their hypertension status were more likely to be female, older, illiterate, widow, former smoker (for hypertension), nonsmoker (for awareness), hookah user (for awareness), overweight or obese and in lower economic status. Those who use hookah and were drug abuser were less likely to have hypertension. Drug and alcohol users were less likely to have awareness regarding their condition. In addition, those received treatment were more likely to be female, older, widow, overweight or obese or being in 5^th^ quantile of wealth index. The results for having a controlled blood pressure were similar with other component in terms of sex and wealth index. Females and people with better wealth index and those with better education were more likely to be under control of anti-hypertensive treatment. However, older people, former smoker, hookah and alcohol user and drug abuser were less likely to have controlled blood pressure (Table 2).

**Table 2:** Univariate and multivariate odds ratio for prevalence and ATC of hypertension in the PERSIAN study^a b^

Variables		HTN		Awareness		Treatment		Controlled	
		Crude OR(95%CI)	Adjusted OR(95%CI)	Crude OR(95%CI)	Adjusted OR(95%CI)	Crude OR(95%CI)	Adjusted OR(95%CI)	Crude OR(95%CI)	Adjusted OR(95%CI)
Sex(Ref:male)	Female	1.49(1.44,1.54)	1.31(1.25,1.36)	3.35(3.13,3.59)	3.68(3.37,4.03)	1.20(1.07,1.34)	1.44(1.26,1.63)	1.41(1.31,1.52)	1.69(1.55,1.84)
Age (Ref:35–39 years)	40–44	1.89(1.72,2.07)	1.80(1.65,1.98)	1.70(1.42,2.03)	1.44(1.19,1.74)	1.34(1.05,1.72)	1.38(1.07,1.77)	0.97(0.75,1.26)	0.97(0.75,1.26)
	45–49	3.44(3.16,3.75)	3.24(2.97,3.53)	2.57(2.17,3.04)	2.14(1.78,2.56)	1.89(1.49,2.39)	2.03(1.60,2.58)	0.90(0.71,1.14)	0.91(0.71,1.16)
	50–54	6.12(5.64,6.64)	5.78(5.31,6.29)	3.56(3.02,4.20)	3.07(2.57,3.67)	2.74(2.17,3.46)	3.03(2.38,3.85)	0.86(0.68,1.09)	0.90(0.71,1.14)
	55–59	9.16(8.44,9.95)	9.01(8.28,9.81)	4.49(3.81,5.28)	4.37(3.65,5.24)	3.42(2.71,4.32)	3.98(3.13,5.07)	0.79(0.62,0.99)	0.86(0.68,1.09)
	> 59	15.05(13.89,16.31)	15.67(14.39,17.07)	5.36(4.58,6.27)	5.88(4.92,7.03)	5.03(4.0,6.33)	6.30(4.93,8.05)	0.76(0.60,0.95)	0.89(0.71,1.13)
Education (Ref:illiterate)	1–5 y	0.51(0.48,0.53)	0.90(0.86,0.94)	0.66(0.60,0.73)	1.05(0.94,1.18)	0.71(0.62,0.81)	1.0(0.86,1.15)	1.17(1.07,1.27)	1.14(1.03,1.25)
	6-8y	(0.40,0.37)	0.91(0.84,0.96)	0.44(0.39,0.49)	1.0(0.89,1.17)	0.85(0.72,0.99)	1.26(1.03,1.33)	1.14(1.03,1.33)	1.19(1.03,1.35)
	9-12y	0.35(0.0.32,0.38)	0.88(0.83,0.93)	0.41(0.37,0.45)	1.13(0.98,1.31)	0.81(0.68,0.99)	1.41(1.12,1.77)	1.24(1.08,1.40)	1.23(1.07,1.42)
	≥ 13 y	0.30(0.28,0.32)	0.83(0.78,0.90)	0.38(0.34,0.43)	1.20(1.0,1.44)	0.80(0.65,1.0)	1.37(1.05,1.79)	1.29(1.11,1.49)	1.29(1.07,1.54)
Marital status(Ref:married)	single	0.38(0.32.0.45)	0.84(0.71,1.0)	0.28(0.20,0.39)	0.34(0.24,0.48)	0.41(0.25,0.65)	0.59(0.36,0.96)	0.72(0.46,1.13)	067(0.43,1.05)
	Widow	2.20(2.08,2.33)	1.11(1.04,1.19)	2.56(2.24,2.92)	1.21(1.03,1.44)	1.40(1.17,1.68)	1.02(0.84,1.24)	1.16(1.04,1.29)	1.16(1.03,1.30)
Hookah(Ref:No)	Yes	0.91(0.86,0.97)	1.14(1.07,1.23)	2.94(2.84,3.04)	0.90(0.78,1.03)	1.11(0.89,1.39)	1.20(0.96,1.51)	0.85(0.74,0.97)	0.89(0.77,1.02)
Drug abuse(Ref:No)	Yes	0.82(0.77,0.86)	1.04(0.97,1.11)	0.55(0.49,0.61)	1.03(0.91,1.17)	1.34(1.07,1.67)	1.51(1.20,1.91)	0.68(0.60,0.77)	0.82(0.72,0.93)
Alcohol(Ref:No)	Yes	0.68(0.63,0.73)	1.07(0.98,1.16)	0.44(0.39,0.50)	0.96(0.82,1.12)	0.86(0.66,1.11)	1.04(0.79,1.37)	0.78(0.66,0.93)	0.92(0.77,1.11)
Smoking (Ref: Non-smoked)	Current	0.57(0.54,0.60)	0.75(0.70,0.80)	0.48(0.43,0.53)	1.21(1.06,1.37)	0.83(0.69,0.99)	0.98(0.80,1.21)	0.98(0.86,1.12)	1.33(1.15,1.54)
	Former	1.26(1.19,1.33)	1.10(1.03,1.18)	0.57(0.51,0.63)	1.17(1.03,1.34)	1.12(0.92,1.36)	1.09(0.87,1.35)	0.80(0.71,0.90)	1.08(0.94,1.23)
BMI (ref: > 25)	25.0–29.9	1.77(1.69,1.86)	1.93(1.84,2.03)	1.53(1.40,1.68)	1.32(1.18,1.47)	1.15(0.99,1.34)	1.19(1.02,1.39)	1.07(0.96,1.19)	0.98(0.88,1.09)
	30.0–34.9	2.56(2.44,2.69)	2.77(2.62,2.92)	2.03(1.84,2.24)	1.43(1.27,1.61)	1.23(1.05,1.44)	1.31(1.10,1.54)	1.08(0.97,1.21)	0.92(0.82,1.03)
	≥ 35	3.69(3.46,3.92)	4.05(3.78,4.34)	2.61(2.30,2.96)	1.41(1.22,1.64)	1.23(1.01,1.49)	1.36(1.11,1.66)	0.97(0.85,1.10)	0.77(0.68,0.89)
Economic status(Ref:1^ft^ quintile)	2^nd^ quintile	0.88(0.84,0.92)	1.0(0.95.1.06)	0.93(0.84,1.04)	1.27(1.13,1.43)	1.11(0.95,1.29)	1.18(1.0,1.39)	1.15(1.04,1.27)	1.17(1.06,1.30)
	3^rd^ quintile	0.77(0.74,0.81)	0.97(0.92,1.03)	0.90(0.81,1.01)	1.50(1.32,1.70)	1.03(0.88,1.21)	1.17(0.99,1.38)	1.28(1.15,1.42)	1.31(1.17,1.46)
	4^th^ quintile	0.67(0.64,0.71)	0.93(0.87,0.99)	0.71(0.64,0.79)	1.47(1.29,1.67)	0.99(0.85,1.17)	1.18(0.99,1.41)	1.36(1.21,1.51)	1.37(1.21,1.54)
	5^th^ quintile	0.64(0.60.0.67)	0.91(0.85,0.98)	0.77(0.69,0.86)	1.93(1.65,2.27)	1.26(1.05,1.53)	1.44(1.16,1.80)	1.56(1.38,1.77)	1.62(1.40,1.87)

^a^ For all calculations, we used centers as the primary sampling units in the survey design and used probability weights

^b^ Multivariate odds ratio analyzes are adjusted to age, sex, and education

After adjustment for possible confounding variable, People with hypertension were more likely to b female, older, illiterate, hookah user, former smoker, overweight or obese and to be in the first quartile of wealth index. Current smokers were less likely to have hypertension. Similarly, those with better awareness about their hypertension were more likely to be female, older, widow, current or former smoker, participants with BMI and with better wealth index. In addition, those who received treatment were more likely to be female, older, more educated, drug abuser, wealthier and people with higher BMI. However, people with uncontrolled hypertension were more likely to be drug abuser and obese. Wealthier people, current smokers, widows, those with higher education and females were more likely to have controlled hypertension (Table 2).

### The results of socioeconomic inequality

**Fig. 1:**
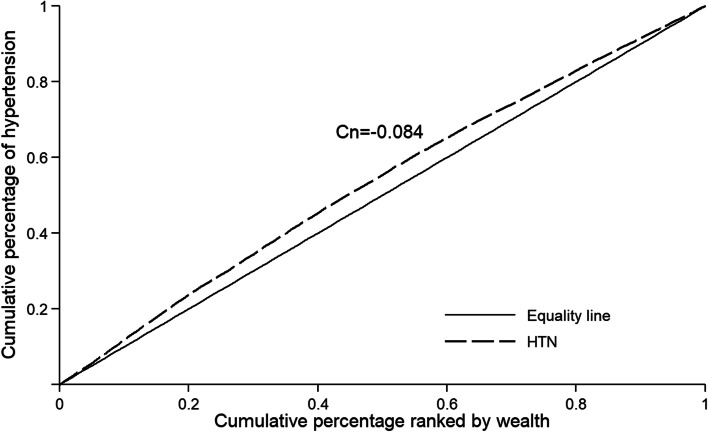
Concentration curve for the prevalence of hypertension in PERSIAN cohort study

The value of the concentration index for prevalence of HTN was equal to -0.084 (95% CI: -0.091; -0.077). The curve lies above the line of equality, indicating that higher prevalence of HTN among the poor population (Fig. 1). Although the results of prevalence of HTN and Cn have not been presented separately for cohort centers, concentration index for prevalence of HTN was negative for all centers. The highest level of inequality was observed in Yazd with a concentration index of -0.23 and the lowest level of inequality was observed in Zahedan with an index of -0.009. The concentration index -0.020 (95% CI: -0.031; -0.010)for men and -0.112 (95% CI: -0.121; -0.103) for women. The concentration index was obtained for awareness -0.022 (95% CI: -0.036; -0.009), treatment 0.023(95% CI: 0.008; 0.037) and control 0.090 (95% CI: 0.076; 0.103.

The Results of the decomposition analysis of SEI in HTN among PERSIAN Cohort participants has been shown in Table 3. The most important contributor to SEI in prevalence of HTN were age (58.46%); followed by SES(32.40%), and being female (6.32%). The BMI had a negative contribution of 21.84%. In total, the variables included in the study explained 68.13% of the SEI in prevalence of HTN.

**Table 3:** Results of the decomposition analysis of SEI in HTN among PERSIAN Cohort participants in Iran

Variables		Elasticity	C_k_	Absolute contribution	Percent contribution	Sum percent contribution
sex (Ref:male)	Female	0.059	-0.090	-0.005	6.32	6.32
Age (Ref = 35–39 years)	40–44	0.031	0.047	0.001	-1.71	58.46
	45–49	0.062	0.066	0.004	-4.90	
	50–54	0.108	0.050	0.005	-6.41	
	55–59	0.165	-0.044	-0.007	8.69	
	> 59	0.280	-0.188	-0.053	62.80	
Education (Ref:illiterate)	1–5 y	0.000	-0.177	0.000	-0.08	-6.42
	6–9 y	0.005	0.070	0.000	-0.42	
	10–12 y	0.008	0.342	0.003	-3.35	
	≥ 13 y	0.003	0.711	0.002	-2.57	
Marital status (Ref:married)	single	-0.001	-0.296	0.000	-0.42	1.22
	Widow	0.004	-0.361	-0.001	1.64	
Hookah(Ref:No)	Yes	0.006	0.126	0.001	-0.93	-0.93
Drug abuse(Ref:No)	Yes	0.011	-0.064	-0.001	0.81	0.81
Alcohol(Ref:No)	Yes	0.004	0.201	0.001	-0.94	-0.94
Smoking status (Ref: Non-smoked)	Current	-0.026	-0.023	0.001	-0.70	-0.94
	Former	0.005	0.043	0.000	-0.24	
BMI (Ref: < 25)	25.0–29.9	0.099	0.056	0.006	-6.63	-21.84
	30.0–34.9	0.137	0.070	0.010	-11.43	
	≥ 35	0.062	0.051	0.003	-3.78	
Economic status (Ref: 1^ft^ quintile)	2^nd^ quintile	-0.003	-0.492	0.001	-1.74	32.40
	3^rd^ quintile	-0.008	0.041	0.000	0.37	
	4^th^ quintile	-0.018	0.579	-0.011	12.72	
	5^th^ quintile	-0.017	1.065	-0.018	21.05	
Total explained				-0.057		68.13
residual				-0.027		31.87
total				-0.084		100

## Discussion

In this study, we extend previous studies in three ways. As well as investigation of factor related to prevalence and ATC of HTN, we measured the inequalities in prevalence of HTN for the first time in a nationwide study. In addition, we explored sources of inequality applying decomposition analysis. This study revealed that the ATC of HTN were 73.74%, 82.22%, and 74.44% in PERSIAN cohort, respectively. Previous studies showed that the trend of awareness, treatment, and control of HTN among Iranian hypertensive people from 2000 to 2019 have been improving [11]. While the awareness of being hypertensive was more than 73% among our population, only less than 53% of Chinese, Malay, and Indian population were aware of their HTN. Controlled HTN was also higher in the PERSIAN Cohort population in comparison to some East Asian counties such as southwestern of China and South Korea (i.e. 10% and 42.1%) [40, 41]. However, SEI may not affect receiving antihypertensive treatment due to affordable medication in Iran [42]. If we consider the PERSIAN cohort population as a proxy of the entire Iranian population, we can argue that a good control of HTN has been achieved in recent decades, more than what has been reported in the best performing countries in control of HTN (less than 70%) [43].The upward trend of control of HTN over recent decade in Iran indicate that conducting the active surveillance program provided by Primary Health Care (PHC) workers and Iranian version of Package of Essential Non-communicable Disease (IraPEN) program worked satisfactorily[44]. As it is well documented previously, in our study, people's awareness of their HTN improved with increasing age. However, in a study conducted in South Korea, younger people were more aware of their HTN [45]. In addition, the prevalence and ATC of HTN in women was higher than that of men in all age groups. These differences between men and women were greater in the older age groups. This difference in prevalence may be due to the estrogen drop in women of menopausal age that has previously been discussed. The effect of lifestyle differences in older women compared to men should be investigated in the future studies [46, 47].

People with higher education had greater awareness, and control of HTN. The results of a study conducted in South Korea showed that with increasing level of education, control of HTN also increased, but no relationship was observed between control of HTN and the level of SES [48]. In terms of treatment, a similar pattern was seen in our study, except in individuals who had more than 13 years of education. This is in line with the previous studies in Iran that showed people with higher levels of education obtained less health care services [49].

Although, individuals with a greater BMI had a higher likelihood of prevalence, awareness, and control of HTN, they have been less likely to control their HTN. It may be due to the higher level of fat mass that leads to increase in salt retention and insulin resistance, and higher level of HTN. The results of our study, in consistent with other studies, showed that increasing BMI increases the likelihood of HTN [50–52].

Higher level of wealth index was significantly associated with lower prevalence of HTN and better treatment and control of this condition. Individuals at the lower SES levels were more likely to be aware of their HTN, but their higher SES counterparts were more likely to have received antihypertensive treatment, and more likely to have controlled HTN. The results of a meta-analysis study showed that the prevalence of HTN is concentrated in groups with lower SES but it is more inconsistent with ATC of HTN [40].

The negative value of concentration index of HTN (-0.084) indicates that the hypertensive individuals in Iran are more concentrated in low SES groups. This result is similar to that of previous study among 690 individuals in Tabriz city, North western of Iran, that showed a negative concentration index of HTN (-0.154). These findings also are in line with previous studies conducted in other countries. If the value of concentration index multiply by 75, we achieve an estimation of the percentage of hypertensive patients to be redistributed from the poorer half to the richer half, to obtain Cn value of zero and a distribution of equality. Therefore, in our study equality in the distribution of HTN can be achieved by redistributing 6.3%. (0.084*75) i.e. about 2,199 of hypertensive population from the poorer half to the richer half.

The decomposition analysis showed that age, economic status, and sex were the key determinants of the pro-rich inequality in the prevalence of HTN. In our study, age was the most important factor in increasing inequality in HTN by 58.46%The concentration index for prevalence of HTN in a study conducted by Si et al.(2017) in China was -0.464. Similar to our findings, age has been the most important factor in explaining the inequality in HTN [53].

Our study showed the economic status has increased SEI in HTN by 32.40%. These results imply that although the PHC are free of charge across Iran, we are still suffering from the imbalanced accessibility and utilization of primary health services between the poor and the rich. These results indicate that mitigating the economic inequality could help decrease the gap in the access to healthcare by improving the healthcare utilization in the poor.

PERSIAN cohort is a large and nationwide study aiming to investigate the incidence of major NCDs and their risk factors in Iran over 15 years of follow-up. All centers used the same questionnairs with the same protocols covering different ethnicities living in Iran which such strategies limit the bias. However, our sample is not a random sample of all Iranian inhabitants and therefore one may generalized our results to the whole country with caution. In addition, due to the cross-sectional design of our study, the reported associations do not represent any causality. While most of the measured variables were objective, our slef-reported measurement regarding the alcohol and substance abuse might not be valid. We categorized these variables as ever and never used.

This study is the first of its type addressing inequalities in HTN in Iran, where there is a very well-known public health network covering remote areas as well as big metropolitan cities. With recent changes, all Iranians currently have an electronic medical record, however, data from these records is not yet available. Therefore, results of our study, using data from a nationwide cohort study including people from different geographical areas and ethnicities with various levels of SES can be an acceptable substitute to estimate the prevalence of HTN as well as its ATC and be used as the basis of future health care and disease prevention policies.

## Conclusion

The prevalence of hypertension was more concentrated among low-SES people with higher level of awareness. However, more concentration of treatment and control of hypertension among people who had higher level of SES indicate that people at higher risk of adverse event of hypertension (low SES group) get less advantage of treatment and control of hypertension. Such a gap between diagnosis (prevalence) and control (treatment and control) of hypertension need to be addressed by public health policymakers.

## Data Availability

The datasets generated during and/or analyzed during the current study are available from the corresponding author on reasonable request.

## Abbreviations

HTN: Hypertension
ATC: Awareness, Treatment and Control
PERSIAN: The Prospective Epidemiological Research Studies in IrAN
Cn: Concentration index(Normalized)
SDGs: Sustainable Development Goals
SEI: SocioEconomic Inequality
SES: SocioEconomic Status
NCD: Non-Communicable Diseases
